# Producing sons reduces lifetime reproductive success of subsequent offspring in pre-industrial Finns

**DOI:** 10.1098/rspb.2007.1051

**Published:** 2007-09-25

**Authors:** Ian J Rickard, Andrew F Russell, Virpi Lummaa

**Affiliations:** Department of Animal and Plant Sciences, University of SheffieldSheffield S10 2TN, UK

**Keywords:** evolutionary ecology, intergenerational cost of reproduction, lifetime reproductive success, Lack's hypothesis, Trivers–Willard hypothesis

## Abstract

Life-history theory states that reproductive events confer costs upon mothers. Many studies have shown that reproduction causes a decline in maternal condition, survival or success in subsequent reproductive events. However, little attention has been given to the prospect of reproductive costs being passed onto subsequent offspring, despite the fact that parental fitness is a function of the reproductive success of progeny. Here we use pedigree data from a pre-industrial human population to compare offspring life-history traits and lifetime reproductive success (LRS) according to the cost incurred by each individual's mother in the previous reproductive event. Because producing a son versus a daughter has been associated with greater maternal reproductive cost, we hypothesize that individuals born to mothers who previously produced sons will display compromised survival and/or LRS, when compared with those produced following daughters. Controlling for confounding factors such as socio-economic status and ecological conditions, we show that those offspring born after elder brothers have similar survival but lower LRS compared with those born after elder sisters. Our results demonstrate a maternal cost of reproduction manifested in reduced LRS of subsequent offspring. To our knowledge, this is the first time such a long-term intergenerational cost has been shown in a mammal species.

## 1. Introduction

Life-history theory states that reproductive events confer costs upon parents (Roff 1992). While studies of wild populations of animals have shown that reproduction is associated with reduced survival, attractiveness and/or future reproductive capacity for breeders, such evidence is by no means universal (Clutton-Brock 1991). Such a lack of evidence for reproductive costs is commonly attributed to high-quality individuals or those breeding in favourable circumstances being ‘immune’ to reproductive costs. However, because a parent's fitness depends not only on its own lifetime fecundity, but also that of its offspring, costs may be inter- as well as intragenerational, particularly in species that are expected to favour their own residual reproductive value at the expense of their offspring (Drent & Daan 1980).

In mammals as well as birds, several studies have tested the hypothesis (Lack 1947, 1948*a*, *b*) that the growth and survival of offspring is compromised in those whose parents provisioned artificially enlarged broods or litters, with equivocal results (Hare & Murie 1992; Mappes *et al.* 1995; Kunkele 2000). However, most studies that have tracked the effects of decreased offspring investment on the success of those offspring have only done so as far as independence or recruitment age. The intergenerational cost of reproduction will remain elusive to such short-term observation if the costs of reduced parental investment are not apparent until the offspring's reproductive life. Only a few studies have investigated parental costs in terms of the reproductive performance of their offspring (Gustafsson & Sutherland 1988; Blondel *et al.* 1998; Koskela 1998; Naguib *et al.* 2006). Perhaps due largely to temporal and logistic constraints of field studies or to the problem of natal dispersal, none has investigated the costs in terms of offspring's lifetime reproductive success.

In this study, we investigate for the first time intergenerational costs of reproduction, measured in terms of offspring's lifetime reproductive success (LRS). We avoid the above logistic problems by using a large pedigree dataset of pre-industrial Finns, which allows following the reproductive events of all offspring throughout their life (see §2). We focus on a differential in cost between individual reproductive events in females and follow the consequent survival and LRS of their subsequent offspring. The differential reproductive cost we use is that of producing male over female offspring. In several mammal species, including humans, there is clear evidence that producing sons is more costly to mothers than producing daughters (table 1). While evidence for a higher cost of males is not ubiquitous (Hewison & Galliard 1999), this has so far manifested itself in those producing sons as greater parasitic load (Festa-Bianchet 1989), delay in return to oestrus (Hogg *et al.* 1992), reduced probability of future reproduction (Clutton-Brock *et al.* 1981), reduced post-partum weight accumulation (Birgersson 1998) and reduced maternal longevity (Helle *et al.* 2002). In addition, in both bighorn sheep, *Ovis canadensis* (Bérubé *et al.* 1996), and humans (Trotnow *et al.* 1976; Magnus *et al.* 1985; Blanchard & Ellis 2001; Côté *et al.* 2003), the offspring born immediately following a son may have lower birth weight than those born after a female. Although birth weight itself may be only a weak predictor of reproductive success in the bighorn sheep (Festa-Bianchet *et al.* 2000), in humans it has long been known to be associated with early survival (Karn & Penrose 1952) and health in adulthood (Barker 1998) and has also been linked to reproductive success among men (Phillips *et al.* 2001; Vågerö & Modin 2002). Thus, there exists in humans a clear precedent for the development of offspring to be affected by the sex of the offspring an individual's mother has produced in the previous reproductive event.

Consequently, we investigate the relationship between the sex of the offspring produced in one reproductive episode and the survival and LRS of offspring produced immediately after, using 521 mothers (hereafter referred to as P_1_) and 1765 of their male and female offspring (hereafter referred to as F_1_) born during the eighteenth and nineteenth centuries in Finland. We examined whether elder sibling sex affected in F_1_ individuals: (i) the probability of surviving to adulthood and (ii) LRS. In addition, we examined (iii) the mechanism through which elder sibling sex affected LRS, namely through lifetime fecundity and/or survival of offspring (F_2_).

## 2. Material and methods

### (a) Demographic data

To investigate how producing sons affects the success of the following offspring in humans, we collected three generations of pedigree data from historical population registers of The Lutheran Church in Finland. Since the seventeenth century, records have been taken of all births, marriages, deaths and inter-parish movements in Finland, making these one of the most reliable sources of demographic data available for a pre-industrial human population. Social class, an important potential confounding factor in studies concerning the allocation of resources between reproduction and self-maintenance (van Noordwijk & Dejong 1986) or production of sons and daughters (Trivers & Willard 1973), is based on the occupation of a husband in the family, which is a good correlate of resources available (Karskela 2001). We categorized each family as poor (e.g. farmless families, servants), middle class (e.g. tenant farmers, smiths, sailors) or wealthy (e.g. priests, officers, farm owners, shipmasters; for details see Pettay *et al.* 2007).

We initially followed the survival and reproductive events of a total of 653 reproductive women (the P generation) from five farming/fishing parishes (Lummaa *et al.* 2007; Pettay *et al.* 2007) born during the years 1709–1815 and recorded full life-history data for their offspring born (the F_1_ generation), as well as birth and subadult mortality data of the F_2_ generation. Here we investigate how the sex of the elder sibling affected the survival and LRS of the F_1_ generation.

All F_1_ individuals were born between the years 1734 and 1861. Consequently, the study period ended before the availability of reliable contraception, freely available healthcare and the associated transition to low mortality and fertility in Finland, which was not complete until the mid-twentieth century (Korpelainen 2003). The mating system was monogamous to an unusually high degree, with 99% of reproductive individuals in our sample being married. Divorce was forbidden, and so remarriage was permitted only in the event of spousal death. Subadult mortality was high, with only 61% of F_1_ individuals in our sample surviving to age 15 (the youngest age of first reproduction recorded in this population). For analysis, we removed cases that did not meet the requirements needed to address the questions of this study (table 2). These were those who were firstborn to their mothers, those who were twins and born to mothers who previously produced twins. We also removed those whose mother had produced more than nine elder siblings previously (4% of remaining cases), because family size is highly skewed and the response terms have high degrees of variance in those of late birth order which decrease model-fitting power. Removal of these cases, plus those for which data were missing, provided a core of 1765 individuals from 521 mothers. Table 3 provides the descriptives relating to the number of individuals in the core data according to their own sex and the sex of their mother's previous offspring.

### (b) Statistical methods

Statistical analyses used to address each of the following three questions were conducted using SAS (SAS Institute Inc., release v. 9.1, 2002–2003). In all analyses (§2*b*(i)–(iii)), parish, year of birth, social class (rich, middle and poor), birth order, interval separating the birth of the focal individual from that of their elder sibling, the total family size (number of F_1_ individuals surviving to age 15) and own sex were entered sequentially into the analyses to control for potential confounding sources of variation. Those confounding terms that were significant were retained and those that were not were dropped from the model. Once the minimal model was found, the sex of an individual's elder sibling (our term of interest) was added to the final model and its significance determined. All two- and three-way interactions involving elder sibling sex were then tested, but none were significant. Finally, the identity of P mothers was fitted as a random term to account for the use of repeated offspring within families. Generalized linear mixed models (GLMMs) were conducted using the GENMOD function in SAS (SAS 1990). All *p*-values are two-tailed and significance levels are set at 0.05.

#### (i) Elder sibling sex and the probability of surviving to age 15

Survival to age 15 (0/1) was considered as a binary response term in a GLMM with logit link function and binomial denominator fixed at 1. After controlling for confounding terms (see §2*b*), we tested the influence of elder sibling sex on the survival probability of all individuals in the core data, 1765 F_1_ offspring delivered by 521 P mothers (1–9 measures per mother).

#### (ii) Elder sibling sex and LRS

LRS was measured as the lifetime number of F_2_ offspring raised to 15 years. This was considered as a continuous response term in a GLMM with Poisson error structure and logarithm link function. Controlling for confounding terms, we tested the influence of elder sibling sex on LRS in a subset of the core data, comprising all those who survived to age 15, who had been followed for their entire reproductive life and those whose social class (an important confounding factor) was known. The end of reproductive lifespan was determined for women as the estimated age of menopause (45 years) and for men as the age by which 90% of men in the whole population had finished reproducing (50 years). This subset comprised 656 individuals, delivered by 322 mothers (1–7 measures per mother). Tables 3 and 4 provide the descriptives of those in this data subset. Since LRS can potentially be negatively influenced by failure to successfully follow all offspring to age 15, the response term was weighted by the proportion of an individual's children whose survivorship to this age was known. Childlessness in this subset (99 individuals, 15%) was associated with the failure to marry among those dying prematurely. Of those who failed to reproduce, less than half (47 individuals, 47%) were married, whereas among those who fathered or gave birth to at least one child, 99% were married.

#### (iii) Elder sibling sex and mechanisms of LRS

Two life-history traits will govern our measure of LRS: (i) lifetime fecundity and (ii) the survivorship of offspring to age 15. Lifetime fecundity was considered as a continuous response term in a GLMM with Poisson error structure and logarithm link function. Controlling for confounding terms, the influence of elder sibling sex on lifetime fecundity was analysed using the same subset of data as used in analysis (§2*b*(ii)).

The proportion of offspring surviving to age 15 was examined by considering LRS as a response term in a GLMM with logit link function and a variable binomial denominator equal to fecundity. After controlling for confounding terms, we tested the influence of elder sibling sex on the survival probability of a F_1_ individual's offspring using a further subset of the data. This was because only those individuals with lifetime fecundity of at least 1 could be included in this analysis. This second subset comprised 557 individuals, delivered by 298 mothers (1–7 measures per mother). Tables 3 and 4 provide the descriptives of those in this data subset. As with analysis (§2*b*(ii)), the response term was weighted by the proportion of an individual's children whose survivorship to age 15 was known.

## 3. Results

### (a) Elder sibling sex and the probability of surviving to age 15

Sixty-one per cent of the offspring in the core data survived to age 15. Survival differed between parishes (*Χ*_4_^2^=36.09, *p*<0.0001) and was negatively associated with birth order (*Χ*_1_^2^=3.75, *p*=0.053) and positively with family size (*Χ*_1_^2^=84.67, *p*<0.001). Those born after a long birth interval (*Χ*_1_^2^=4.46, *p*=0.035) and those born later in the time series (*Χ*_1_^2^=3.44, *p*=0.064) were also more likely to survive. After controlling for these effects, we found no evidence to suggest that survival to age 15 was associated with elder sibling sex (*Χ*_1_^2^=0.00, *p*=0.98; figure 1). Non-significant potential confounding terms were (mother's) social class (*Χ*_2_^2^=0.81, *p*=0.67) and own sex (*Χ*_1_^2^=0.16, *p*=0.69). Owing to collinearity, family size but not birth order was included in the final model.

### (b) Elder sibling sex and LRS

In the subset of data comprising those offspring who survived to age 15 themselves and were successfully followed until the end of potential reproductive life, the median number of children raised to age 15 was 2 and the maximum was 9. A large proportion of individuals (27%) raised no children to adulthood and thus had an LRS of zero. LRS differed marginally between parishes (*Χ*_4_^2^=8.79, *p*=0.067) and was higher for those in the rich and middle social classes than those in the poor class (*Χ*_2_^2^=47.18, *p*<0.0001, adjusted means±s.e. 2.83±0.15, 2.75±0.17 and 1.09±0.15 for rich, middle class and poor, respectively). After controlling for these effects, we found that LRS was significantly higher (27%) in those born after an elder sister than in those born after an elder brother (*Χ*_1_^2^=12.01, *p*=0.0005; figure 2). Finally, LRS was not significantly associated with year of birth (*Χ*_1_^2^=0.28, *p*=0.60), birth order (*Χ*_1_^2^=0.49, *p*=0.48), family size (*Χ*_1_^2^=0.43, *p*=0.51), birth interval (*Χ*_1_^2^=0.01, *p*=0.92) or own sex (*Χ*_1_^2^=0.30, *p*>0.58).

### (c) Elder sibling sex and mechanisms of LRS

Among those who survived to age 15 and were successfully followed until the end of potential reproductive life, the median lifetime fecundity was 4 and the maximum 13. Fecundity differed between parishes (*Χ*_4_^2^=39.12, *p*<0.0001) and was higher for those in the rich and middle social classes than those in the poor class (*Χ*_2_^2^=42.70, *p*<0.0001, adjusted means±s.e. 4.81±0.18, 4.66±0.21 and 2.45±0.28 for rich, middle class and poor, respectively). After controlling for these effects, we found that lifetime fecundity was significantly higher (12%) in those born after elder sisters than in those born after elder brothers (*Χ*_1_^2^=5.06, *p*=0.025; figure 3*a*). Lifetime fecundity was not significantly associated with year of birth (*Χ*_1_^2^=0.11, *p*=0.74), birth order (*Χ*_1_^2^=0.00, *p*=0.96), family size (*Χ*_1_^2^=1.22, *p*=0.27), previous birth interval (*Χ*_1_^2^=0.82, *p*=0.37) or own sex (*Χ*_1_^2^=0.10, *p*=0.75).

After excluding childless individuals, 557 were included in the analysis of offspring survival rate. Only 25% of couples successfully raised all of the children they produced. Offspring survival rate, weighted in the analysis by the proportion of offspring successfully followed, differed between parishes (*Χ*_4_^2^=32.02, *p*<0.001) and was higher for those in the rich and middle social classes than those in the poor class. After controlling for these confounders, there was a non-significant trend towards offspring survival rate being higher for those born after elder female versus male siblings (*Χ*_1_^2^=2.80, *p*=0.094; figure 3*b*). Offspring survival rate was not associated with year of birth (*Χ*_1_^2^=2.68, *p*=0.10), birth order (*Χ*_1_^2^=1.97, *p*=0.16), family size (*Χ*_1_^2^=0.35, *p*=0.55), F_1_ birth interval (*Χ*_1_^2^=2.17, *p*=0.14) or own sex (*Χ*_1_^2^=0.00, *p*=0.97).

## 4. Discussion

Our results show that F_1_ individuals who were born to mothers who had previously produced a son, when compared with those born to mothers who had previously produced a daughter, had similar probability of survival to adulthood but lower LRS. This latter result was mediated primarily through reduced lifetime fecundity of those born after an elder brother. Our results were apparent in all five parishes and across all social classes, indicating that they were not dependent on any particular ecological or social factors (see electronic supplementary material).

We consider three explanations to account for these observations. First, direct sibling interaction could lead to the reduced success of the younger offspring through the elder male siblings having a direct negative influence on the development of their younger siblings. As has been seen in previous studies on humans, elder offspring may influence the survival (Harpending & Pennington 1991), or reproductive success (Mace 1996) of their younger siblings. In particular, sex-specific competition between siblings for resources and reproductive opportunity may be an important determinant of reproductive success (Low 1990; Low & Clarke 1991; Mace 1996; Mulder 1998). Second, the results could be caused by non-independence of the subsequent and previous offspring sex. Maternal condition may simultaneously influence both offspring LRS and the likelihood of producing male versus female offspring, specifically with mothers with greater access to resources being more likely to produce sons (Trivers & Willard 1973). Finally, individuals may have reduced success because their mother producing a son versus a daughter in the previous reproductive event rendered her less able to invest in the next offspring.

The hypothesis that males have a direct influence on their younger siblings predicts that if an elder sibling died before the birth of the focal individual, then there would be no relationship between elder sibling sex and the survival and LRS of the focal individual. Owing to high infant mortality, elder sibling death prior to the birth of the focal individual occurred in at least 30% of the cases in all datasets. It was therefore possible to test this hypothesis by entering into each model an interaction between elder sibling sex and whether the elder sibling died prior to or after the focal individual's birth. This interaction did not approach significance in either survival to age 15 (*Χ*_1_^2^=1.54, *p*=0.21) or LRS (*Χ*_1_^2^=0.32, *p*=0.57). Similarly, the survival of the elder sibling to age 15 did not modify the relationships (*Χ*_1_^2^=1.75, *p*=0.19; *Χ*_1_^2^=0.47, *p*=0.49), pointing out that there was no indication of sex-specific post-natal interactions between siblings affecting our measures of offspring success. Finally, controlling for the completed total family size, which is an overall measure of sibling competition (Parker *et al.* 2002), failed to change the results. This suggests that the relationship of elder sibling sex with LRS and lifetime fecundity was not due to direct interaction between the elder sibling and the focal individual.

The hypothesis that non-random sex allocation might give rise to younger siblings born after sons having reduced success predicts that P offspring sex is influenced by maternal condition, with mothers who have access to a high level of resources being more likely to produce a son than a daughter (Trivers & Willard 1973). However, if maternal condition had influenced offspring sex, we would expect those born to mothers who had previously produced daughters to have lower than average LRS and lifetime fecundity. We would also expect LRS to be lowest in those cases where both the elder sibling and the focal individual were females and highest in those cases where both were males. This is because high-resource mothers would (i) benefit from a male bias in offspring sex allocation and (ii) produce high-quality offspring. However, not only was there no difference in LRS between the sexes but there was also no indication of any interactions between the sex of the focal individual and the elder sibling in these models (survival: *Χ*_1_^2^=0.86, *p*=0.35; LRS: *Χ*_1_^2^=0.01, *p*=0.93; see graphs in the electronic supplementary material). Therefore, non-independence of offspring sex or sex ratio with respect to offspring LRS is unlikely to account for the strong relationship we observe between previous offspring sex and focal offspring LRS.

The simplest explanation for offspring born after sons having reduced reproductive success is that mothers who produced sons incurred a greater reproductive cost, and hence were less able to invest in future reproductive events, with this reduced investment having long-term consequences for her subsequent offspring. This conclusion is also supported by considerable evidence from other mammal studies, showing that producing sons versus daughters is indeed more costly to mothers as well as to subsequent offspring (table 1). Lower LRS and fecundity of those born after sons in this population can therefore be regarded as part of an emerging picture of a sex bias in the cost of offspring to mothers (see §1). For example, previous studies have shown that human offspring born after sons have reduced birth weight compared with those born after daughters (Trotnow *et al.* 1976; Magnus *et al.* 1985; Blanchard & Ellis 2001; Côté *et al.* 2003) and are also smaller as adults (I. J. Rickard 2007, unpublished results). Smaller sizes at birth and in adulthood are in turn related to reduced reproductive success in adulthood (Lummaa & Clutton-Brock 2002), offering one potential mechanism for our findings. As with all long-term correlative studies, special attention should be given to the potential of bias being introduced by the loss of individuals to follow up (Van de Pol & Verhulst 2005). Those individuals who were excluded from the analysis because they lacked specific data may have been affected differently by the sex of the offspring their mother produced previously. However, we consider this possibility unlikely, because these excluded individuals did not appear to differ from those included in terms of their discernible or life-history characteristics. Furthermore, the probability of exclusion from the data subsets was not related to elder sibling sex (comparison of proportions of those with elder male and female siblings between core data and subset 1, *Χ*_1_^2^=0.58, *p*=0.45).

The consistency of the relationship between elder sibling sex and LRS is worth remarking upon. The association was not modified by mother's social class (two-level factor, *Χ*_1_^2^=1.15, *p*=0.29) or the interval separating the birth of the focal individual and their elder sibling (*Χ*_1_^2^=0.78, *p*=0.76). This is intriguing, because, for example, mothers of a high social class would have more access to resources, and therefore would be expected to withstand any higher energetic cost of producing a son over a daughter. Similarly, it would be expected that those mothers who gave birth to the focal individual a long time after the elder sibling would have been better able to recover from the extra cost of producing a son. However, in addition to, or perhaps instead of, their possibly higher demand on limited maternal resources, the cost differential in producing sons may be due to the factors that are independent of the mother's condition. For example, during development, male and female foetuses produce different quantities of hormones (Clark *et al.* 1991). Because these hormones may diffuse across foetal membranes and amniotic fluid, the endocrine profiles of expectant mothers may differ according to the sex of the offspring being carried. A hormone such as testosterone, which occurs in much higher concentration in a male foetus than in a female adult (Meulenberg & Hofman 1991), could interfere directly with a mother's ability to provide for future offspring by compromising her immunocompetence and increasing her susceptibility to costly disease (Klein 2000). This could in turn reduce her ability to invest in subsequent offspring. Another intriguing observation that has implications for evaluating the potential mechanism is that the effect is not conditional upon, or altered by, the sex of the focal individual. (*Χ*_1_^2^=0.01, *p*=0.93). It suggests that, while in polygynous ungulates the early determinants of reproductive success may differ between the sexes (Kruuk *et al.* 1999), in monogamous human societies such as this one, there may be less of a difference. To further understand the mechanism(s) behind our findings, we are currently investigating the relationship between elder sibling sex and those life-history traits that underlie lifetime fecundity.

Regardless of the underlying mechanism, we have shown clear evidence for an effect of previous maternal investment in a son on the LRS and lifetime fecundity of subsequent offspring. This has implications for calculations of maternal fitness and for considering the selective pressures and ecological constraints relevant to the evolution of adaptive sex-ratio adjustment. In polygynous mammals, a prospective mother with access to more resources than her competitors may benefit from producing a son rather than a daughter if she can better use those resources to improve the reproductive success of a son (Trivers & Willard 1973). Conversely, a prospective mother with access to a relatively low level of resources will benefit by producing a daughter, because any son she produces will ultimately lose out in competition for mates with the sons produced by high-resource females (Trivers & Willard 1973). While our results are not at odds with the qualitative predictions of the Trivers–Willard (1973) hypothesis, they suggest that the benefits of such adaptive sex-ratio variation may need to be weighed against additional costs which manifest themselves later, in the attenuated reproductive success of subsequent offspring.

In this study, we have shown, what is to our knowledge, the first evidence of a long-term intergenerational cost of reproduction in a long-lived species. Many studies have tested the effects of manipulated offspring brood size on offspring (Lessells 1986; Hare & Murie 1992; Mappes *et al.* 1995; Kunkele 2000). While failure to find intergenerational costs of enlarged broods may sometimes be due to those costs being expressed within the breeding individual, another reason may be that they are expressed in offspring reproductive performance. Owing to dispersal and practical constraints of field studies, animals from artificially manipulated broods or litters cannot be easily followed into their adult lives. However, the general findings of the small number of studies to have attempted such a long-term approach (Gustafsson & Sutherland 1988; Blondel *et al.* 1998; Koskela 1998; Naguib *et al.* 2006) reinforce the possibility that such unexplored costs of reproduction are widespread. The present study is the first to find a cost of reproduction manifested in reduced LRS of offspring. Observational studies of long-term pedigree data such as this one provide a useful means with which to approach key life-history questions that cannot conveniently be answered with many vertebrate study systems.

## Figures and Tables

**Figure 1:**
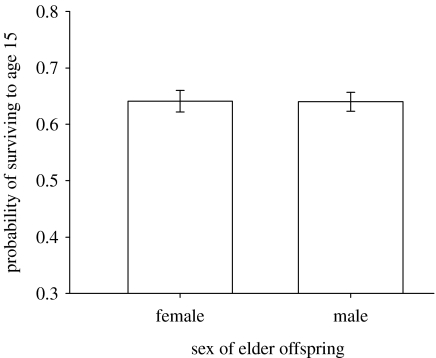
Probability of surviving to adulthood (age 15) according to the sex of elder offspring (mean±s.e.). Values are adjusted means from the final model.

**Figure 2:**
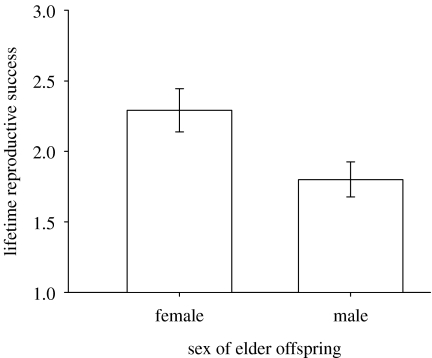
Lifetime reproductive success (number of children raised to age 15) according to the sex of elder offspring (mean±s.e.). Values are adjusted means from the final model.

**Figure 3:**
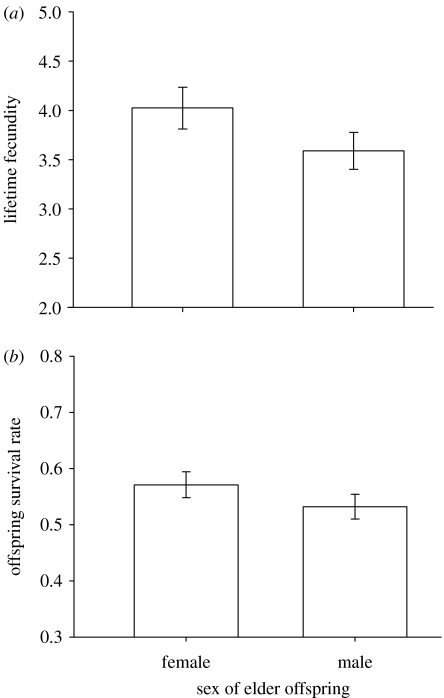
(*a*) Lifetime fecundity (number of children produced) according to sex of elder sibling (mean±s.e.). (*b*) Offspring survival rate (proportion of offspring surviving to age 15) according to the sex of elder offspring (mean±s.e.). Values are adjusted means from the final model.

**Table 1:** Examples of evidence in mammals for higher cost of sons over daughters.

species	nature of cost demonstrated	study
bighorn sheep, *Ovis canadensis*	ewes had higher faecal lungworm count after producing a son	Festa-Bianchet (1989)
	ewes experienced a delay in return to oestrus after producing a son	Hogg *et al.* (1992)
	ewes more likely to have a daughter after producing a son	Bérubé *et al.* (1996)
	ewes produced lighter offspring after producing a son	Bérubé *et al.* (1996)
red deer, *Cervus elaphus*	hinds less likely to reproduce in the year following a son; delayed calving in those who did	Clutton-Brock *et al.* (1981)
	lower post-reproductive maternal survival after weaning a son (subordinate hinds only)	Gomendio *et al.* (1990)
	milk composition dependent on sex, e.g. higher protein percentage in milk provisioned for sons	Landete-Castillejos *et al.* (2004)
fallow deer, *Dama dama*	lower maternal weight accumulation during the period after gestation of offspring, when a son is produced (old hinds only)	Birgersson (1998)
humans	birth interval longer after the birth of a son (low-parity women only)	Mace & Sear (1997)
	maternal longevity associated negatively with the number of sons produced	Helle *et al.* (2002)
	lower birth weight of offspring produced after sons	Trotnow *et al.* (1976); Magnus *et al.* (1985); Blanchard & Ellis (2001); Côté *et al.* (2003)
	higher maternal energy intake in women carrying sons	Tamimi *et al.* (2003)

**Table 2:** Sequence of criteria for the inclusion of F_1_ data and corresponding sample sizes.

criteria for inclusion	no.	use in analysis
all F_1_ generation	4515	
not firstborn	3811	
not a twin	3315	
elder sibling not a twin	3100	
survival to age 15 known	2769	
lifespan of elder sibling known	1862	
elder sibling sex known	1854	
data available for potential confounders	1842	
birth order <11	1765	core data: analysis (i)
survived to age 15	1081	
tracked for reproductive lifespan	716	
social class known	656	subset 1: analyses (ii), LRS and (iii), fecundity
produced at least one child	557	subset 2: analysis (iii), offspring survival rate

**Table 3:** Number of individuals according to own sex and elder sibling sex in the core data, subset 1 and subset 2.

		elder sibling female	elder sibling male	total
core data	focal individual female	407	454	861
	focal individual male	401	503	904
	total	808	957	1765
subset 1	focal individual female	141	161	302
	focal individual male	171	183	354
	total	312	344	656
subset 2	focal individual female	124	137	261
	focal individual male	146	150	296
	total	270	287	557

**Table 4:** Descriptive statistics of reproductive parameters of those F_1_ offspring who survived to age 15 themselves and were successfully followed until the end of potential reproductive life (data subset 1), split by sex and social class (all mean±1 s.d.). (Lifespan here thus refers to the total number of years lived by those who survived to adulthood (age 15).)

social class	sex	total *N*	lifespan	age at marriage	age at first reproduction	age at last reproduction	lifetime fecundity	number of surviving offspring
rich	m	178	54.82±16.30	26.83±5.40	27.85±5.45	39.78±8.32	4.81±3.26	2.47±2.11
	f	140	61.03±16.27	24.73±5.09	25.52±4.59	38.37±5.10	5.09±3.05	2.94±2.26
middle	m	118	58.82±16.42	27.62±5.11	29.06±6.98	41.44±8.27	4.81±3.06	2.66±2.39
	f	108	63.76±16.97	27.11±6.30	27.27±5.69	38.60±6.20	4.51±2.99	2.48±1.94
poor	m	58	48.42±18.42	27.21±5.40	27.48±5.11	37.95±8.88	2.41±3.02	1.17±1.63
	f	54	52.50±19.34	28.45±6.94	28.52±5.70	35.40±6.13	2.11±2.11	0.96±1.37
all	m	354	54.41±16.89	27.15±5.29	28.25±6.03	40.19±8.40	4.42±3.27	2.32±2.19
	f	302	60.50±17.49	26.17±5.98	26.63±5.30	38.01±5.77	4.35±3.07	2.42±2.13

## Supplementary Material

life-history traits according to sex of both elder sibling and focal individual Probability of (*a*) survival to age 15, (*b*) lifetime reproductive success, (*c*) lifetime fecundity and (*d*) offspring survival rate, according to sex of focal individual and sex of elder sibling. All±1 s.e. White bars represent those whose mother previously produced a son and hatched bars represent those whose mother previously produced a daughter. The figures illustrate that the results are similar for male and female focal individuals

lifetime reproductive success according to elder sibling sex and parish Lifetime reproductive success (mean±1 s.e.) according to sex of elder sibling for the five different study parishes. White bars represent those whose mother previously produced a son and hatched bars represent those whose mother previously produced a daughter. The figure illustrates that the difference is consistent across all of the studied populations
