# Supplementary material for: Producing sons reduces lifetime reproductive success of subsequent offspring in pre-industrial Finns
Source: Proc Biol Sci. 2007 Sep 25;274(1628):2981–8. doi: 10.1098/rspb.2007.1051 (PMC2211518; doi:10.1098/rspb.2007.1051)
Supplement: Supplementary file 1 [file rspb20071051s06.doc]

Supplementary figure 1: Probability of survival to age 15 (a), lifetime reproductive success (b), lifetime fecundity (c) and offspring survival rate (d) according to sex of focal individual and sex of mother’s previous offspring. All ± 1 standard error. White bars represent those whose mother previously produced a son and hatched bars represent those whose mother previously produced a daughter. The figures illustrate that the results are similar for male and female focal individuals.

Elder sibling female

Elder sibling male
