# Supplementary material for: Producing sons reduces lifetime reproductive success of subsequent offspring in pre-industrial Finns
Source: Proc Biol Sci. 2007 Sep 25;274(1628):2981–8. doi: 10.1098/rspb.2007.1051 (PMC2211518; doi:10.1098/rspb.2007.1051)
Supplement: Supplementary file 2 [file rspb20071051s07.doc]

Supplementary figure 2: Lifetime reproductive success (mean ± 1 standard error) according to sex of elder sibling for the five different study parishes. White bars represent those whose mother previously produced a son and hatched bars represent those whose mother previously produced a daughter. The figure illustrates that the difference is consistent across all of the studied populations.

Elder sibling male

Elder sibling female
